# 

**Published:** 1890-02

**Authors:** 


### CONTENTS.

### *EDITORIALS.*

| | PAGE |
|---|---|
| La Grippe, | 49 |
| Wines and Liquors in Medicine, | 51 |
| Mercurius in Typhoid Fever, | 51 |
| Antiseptics, | 52 |
| Microbes, Bacilli, Cocci, | 53 |
| Hahnemannian Homœopathy, | 53 |
| Pathology, | 55 |
| Physiology, | 56 |
| Quinine, | 57 |
| Strong Medicine, | 57 |
| Progressive Homœopathy, | 58 |
| To Mongrels and Allopaths, | 59 |

### *MISCELLANEOUS.*

| | |
|---|---|
| Of the Drug Curative. P. P. Wells, M. D., | 60 |
| Address before the Hahnemann Club of Terre Haute. W. H. Baker, M. D., | 63 |
| Clinical Notes. Dr. Filipe Ascot de Tortosa, | 66 |
| Annual Report and Election by the Managers of the Women's Homœopathic Hospital of Penna., | 70 |
| On Mentagra and Tinea Capitis (Favus). S. L., | 73 |
| The Vital Necessity for Fresh Air in Consumption. Edmund J. Lee, M. D., Phila., | 77 |
| Notes from Past Meetings of The Lippe Society, | 79 |
| Mania. G. W. Sherbino, M. D., | 82 |
| A Synopsis of a Case of Hæmorrhoids, | 83 |
| Verifications of Medorrhinum. G. W. Sherbino, M. D., | 84 |
| La Grippe—Three Cases. H. B. Stiles, M. D., | 86 |
| La Grippe. S. W. Cohen, M. D., | 87 |
| Poisoning by Antipyrin. E. W. Berridge, M. D., | 89 |
| Laryngismus Stridulus. Ella M. Tuttle, M. D., | 91 |
| Sunstroke—Asthma. Wm. Steinrauf, M. D., | 92 |
| Two Clinical Cases. W. A. Yingling, M. D., | 93 |
| Morphine? No! No! No! M. A. A. Wolff, M. D., | 94 |

### *BOOK NOTICES.*

| | |
|---|---|
| Sanitary Entombment, | 94 |
| Medical Topics, | 95 |
| Eating for Strength, | 95 |
| Fifth Annual Report of Trustees of Westborough Insane Hospital, | 95 |
| A Treatise on Materia Medica. By John V. Shoemaker, M. D., | 95 |
| The Trained Nurse, | 96 |
| Transactions of the Massachusetts Homœopathic Medical Society, | 96 |
| Essay on Medical Pneumatology. By J. N. Demarquay, | 96 |
| Congestion of Lungs and its Dangers. By Thomas Nichol, M. D., | 96 |

